# Trunk and craniofacial asymmetry are not associated in the general population: a cross-sectional study of 1029 adolescents

**DOI:** 10.1186/s40001-017-0280-y

**Published:** 2017-09-29

**Authors:** Chiara Arienti, Jorge Hugo Villafañe, Sabrina Donzelli, Fabio Zaina, Riccardo Buraschi, Stefano Negrini

**Affiliations:** 1 IRCCS Don Carlo Gnocchi Foundation, P.le Morandi, 8, 20121 Milan, Italy; 2 ISICO, Italian Scientific Spine Institute, Milan, Italy; 3 Department of Clinical and Experimental Sciences, University of Brescia, Brescia, Italy

**Keywords:** Spinal posture, Trunk asymmetry, Craniofacial morphology, Sagittal posture

## Abstract

**Background:**

The literature did not show clearly if a correlation between trunk and facial asymmetry exists. The aim of this study was to verify the association between trunk and facial asymmetries, and trunk and facial sagittal configuration in adolescents.

**Methods:**

This is a cross-sectional screening study. It was carried out in a small town in Northern Italy, from February to April 2014. Healthy children met the inclusion criteria. Exclusion criteria were subjects with physical and cognitive disability, genetic disease, and polymorphism. All subjects underwent a three phases for postural screening program.

**Results:**

1029 healthy children were 491 females and 538 males with mean age: 12 (range 11–16) years. The association of facial and trunk asymmetry had a point prevalence rate around 1% for the various regions of the spine, the association on the sagittal plane of almost 1.3% for hyperkyphosis and hyperlordosis. Overall, results showed a very low sensitivity, specificity, and predictive values of facial anomalies for trunk asymmetry and sagittal spinal posture.

**Conclusion:**

While correlations between jaw position and body posture for cervical spine can exist, our study denied association with trunk and back in a general population: postural compensatory mechanism may have minimized the effects of one area on the other, if any existed.

## Background

Trunk asymmetry (TA) is a common phenomenon at adolescence and can be considered the clinical presentation of scoliosis [1, 2]. TA also has been shown to predict future scoliosis in children but it is not clear if prevalence of TA is linked with peak of growth in adulthood [3]. The diagnosis of idiopathic scoliosis is based on the detection of an angle of trunk rotation (ATR) in forward bending position (FBP) [4], with cut-off of 7°, the most useful for scoliosis detection [4–7]. Lower levels of ATR are considered to define TA (ATR > 4°) [8, 9]. This is the primary screening procedure and an early diagnosis of TA could reduce spinal deformity surgeries and consequently, the cost-effectiveness [7]. Correlation between bone deformities of the head and trunk has been advocated by some for years, such as the position of spine can influence the craniomandibular system and viceversa [10–12], but never proven [13]. Some studies have been performed with different means, but usually on small populations due to the invasive methodologies required [14]. Nevertheless, a bone deformity causes external signs that can be checked through tools that were developed for screening purposes [6, 15] and that could be applied in large populations [16].

Recently, TA has been systematically studied [17] and an asymmetric growth pattern was found for the entire thoracic area, for the vertebral body, as for the neurocentral junction at different ages in a cohort of 199 non-scoliotic children investigated through CT scan. During human growth, the mechanical load on the spine changes due to motor development and physical changes (stature, weight, and shift in body proportions). An a-synchronicity of this process was hypothesized as a possible etiological factor of idiopathic scoliosis [18]. A relationship between preexistent rotation and organ anatomy was previously demonstrated [19].

Abnormal body posture has long been hypothesized to be responsible of various craniofacial orthopedic and orthodontic conditions, but scientific literature is not able yet to support these assumptions [20]. Pathological curvatures induce in some cases the formation of compensatory curvatures elsewhere along the spine and may result also in compensatory head posture. Therefore, non-physiological curvatures in the frontal axis may cause tilting of the head to either side, whereas curvatures in the sagittal axis may result in forward or backward tilts [21, 22]. Previous studies reported that spine and facial sagittal configurations are related: subjects with a long face tended to have longer and straighter cervical vertebrae, whereas those with a short face have more curved cervical vertebra [11].

The literature does not show clearly if a correlation between trunk and facial asymmetry in sagittal plane configurations exists. So, the aim of the present study was to verify the association between trunk and facial configuration in sagittal and coronal planes, screening a large population of healthy adolescents.

## Methods

### Participants

Recruitment was done in a secondary school with students’ mean age 12 (range 11–16) years, located in Brescia, Italy, from February to April 2014. We considered all the school classes to maintain a similar distribution of ages and genders. We screened a total of 47 school classes. Exclusion criteria were subjects with disability, or any other associated pathology.

### Study design

This is a cross-sectional study performed within a scoliosis school screening program.

The evaluation was based on a postural examination divided in three phases, performed once by two different physiotherapist examiners that used the same protocol of evaluation [23].

Phase 1: collecting demographic and clinical characteristics and previous postural exercise or orthodontic therapy.

Phase 2: clinical evaluation of the coronal and transverse plane. The evaluation was performed from the back side of the subjects that maintained the same position: natural upright posture with feet together, extension of the knees and hands at their sides. In the sagittal plane, the plumbline distance of C7, L3, and S1 from the apex of kyphosis was measured and the Sagittal Index C7 + L3 calculated [24]. The transverse plane was evaluated through Adam’s test: the subject was required to bend the spine forward, while standing with knee extended and both arms and head totally relaxed. The presence of a prominence was assessed with a scoliometer (an instrument able to measure in degrees the ATR).

Phase 3: we took four face photos (two in the coronal plane and two in sagittal plane) with upright subject with feet together, in knee extension and arms at their sides, without dental contact. Then, an oro-maxillo-facial surgeon evaluated all the pictures, to define the facial asymmetry and facial morphology in the coronal and sagittal plane.

### Outcome measurements

Patients were evaluated for trunk and facial asymmetry through clinical assessment.

Trunk Asymmetry with ATR was measured through a Bunnell Scoliometer that evaluated presence of rib prominence and scoliosis convexity [25]. Normal values are ATR = 0° Bunnell, ATR ≥ 4° Bunnell is considered trunk asymmetry, ATR ≥ 7° Bunnell is considered scoliosis [6].

The evaluation of sagittal plane decompensation was made using frontal plane decompensation with plumbline distance of S1 from inter-gluteus cleft above measurement error of 1 cm [26], sagittal trunk morphology with plumbline distances in terms of Sagittal Index C7 + L3 (cut-off 95 mm), and L3 (cut-off 50 mm) and S1. Facial asymmetry was evaluated through clinical assessment and photography, while transverse asymmetry used an actual size (1 × 1) clinical photo [27]. Reference Lines were drawn connecting each external canthus and lip commissure, designated as horizontal reference line (HRL) and lip line (LL), respectively. The soft tissue midsagittal line (STML) was set as the line perpendicular to the HRL, passing through the center of both midpoint of pupil (Pm) and the ridge of the nose. Distance from reference lines was considered [28].

Sagittal morphology was measured through visual inspection of the entire face, palpation to differentiate soft tissue and bony defects, comparison of the dental midline with the facial midline, inspection of symmetry between the bilateral gonial angle and mandibular body lower border. The profile as convex, plain, or concave was defined [28].

### Statistical analysis

Data were analyzed using SPSS version 21.0 (SPSS Inc, Chicago, IL, USA). The results were expressed as means, standard deviations, and/or 95% confidence intervals. Sensitivity, specificity, positive (PPV) and negative predictive values (NPV), accuracy, and positive likelihood ratio (LR = sensitivity/1-specificity) of each maneuver to detect facial and trunk asymmetry was calculated using a 2 × 2 table. PPV was used to check the probability that in case of a craniofacial positive test the subject really had the corresponding trunk morphology (asymmetry or sagittal plane morphology). In all analyses, *p* < 0.05 was considered statistically significant.

The sample size was calculated. The literature shows that only 1.6% of high-school students have a completely symmetric posture [29]. The commonly reported prevalence of idiopathic scoliosis is between 2 and 4% [4], while that of TA was 91% in the study by Nissinen [30] and around 70% according to Burwell et al. [31]. Considering alpha = 5% and a power of 80%, we estimated a sample size of a minimum of 1000 subjects with TA. We included all the subjects of the school classes of one secondary school whose total composition allowed to reach the required power size.

## Results

The overall characteristics of the included population are reported in Table 1.

**Table 1:** Baseline demographics

Variable	Participants, (*n* = 1029)
Demographics characteristics	
Age (years)	12.4 ± 0.9
Gender, female: male [n (%)]	491 (47.7%):538 (52.30%)
Weight (m)	49.0 ± 11.0
Height (m)	157.4 ± 10.2
Predominant side	
Write, right [n (%)]	923 (89.7%)
Throw, right [n (%)]	952 (92.5%)
Foot, right [n (%)]	905 (87.9%)
Eyes, right [n (%)]	768 (74.6%)
Trunk asymmetric (> 4°)	
Thoracic	53 (5.2%); 4.8 ± 1.1
Thoracolumbar	81 (7.9%); 5.3 ± 1.6
Lumbar	74 (7.2%); 5.1 ± 1.5
Trunk asymmetric (> 7°)	
Thoracic	6 (0.6%)
Gender, female [n (%)]	2 (0.4%)
Thoracolumbar	13 (1.3%)
Gender, female [n (%)]	10 (2.0%)
Lumbar	9 (0.9%)
Gender, female [n (%)]	8 (1.6%)
Facial sagittal morphology	
Flat [n (%)]	512 (49.8%)
Convex [n (%)]	215 (42.1%)
Concave [n (%)]	18 (1.7%)
Biprotuso [n (%)]	19 (1.8%)
Harmonious [n (%)]	47 (4.6%)
Facial asymmetric	
Female [n (%)]	215 (20.9%)
Male [n (%)]	197 (19.1%)
Age	
11 years, female [n (%)]	101 (49.5%)
12 years, female [n (%)]	169 (45.3%)
13 years, female [n (%)]	167 (50.3%)
14 years, female [n (%)]	54 (45.0%)

Data are expressed as means ± standard deviations (SD)

Out of the 1029 subjects included in our sample, 96.1% of boys and 93.5% of girls were symmetric (ATR ≤ 3°) in the thoracic region; for thoracolumbar and lumbar regions, the corresponding percentages were 94.6–89.4 and 96.1–89.2%, respectively. The total percentage of scoliosis (ATR > 7°) was 2.72%.

ATR mean values in the participants with a TA were 4.8 ± 1.1, 5.3 ± 1.6, and 5.1 ± 1.5 in the thoracic, thoracolumbar, and lumbar spine, respectively, as shown in Table 1. No significant differences were detected between subjects with or without a facial and trunk asymmetry with respect to sex.

The association of facial and trunk asymmetry was estimated to have a point prevalence rate of 0.90, 0.61, and 1.38% for thoracic, thoracolumbar, and lumbar curves, respectively. There is no possibility to consider the facial asymmetry as a predictor of trunk asymmetry and viceversa, due to the very low sensitivity, specificity, positive and negative predictive values, and likelihood ratios, as shown in Table 2.

**Table 2:** Physical examination findings in facial asymmetry

Result	2 × 2 table		Sensitivity	Specificity	PPV	NPV	OR (95% CI)	+LR (95% CI)	−LR (95% CI)
Trunk asymmetry	TP	FP							
	TN	FN							
Thoracic	20	33	37.7	59.8	4.9	94.7	0.9 (0.51–1.6)	0.94 (0.56–1.34)	1.04 (0.84–1.294)
	392	584							
Thoracolumbar	24	57	29.6	59.1	5.8	91.0	0.61 (0.37–1.0)	0.74 (0.52–1.04)	1.18 (1.01–1.37)
	388	560							
Lumbar	35	39	47.3	60.5	8.5	93.7	1.38 (0.86–2.21)	1.2 (0.93–1.54)	0.87 (0.7–1.01)
	377	578							

*T* true, *F* false, *P* positive, *N* negative, *PPV* positive predictive value, *NPV* negative predictive value

The concerns for the facial sagittal morphology, the clinical diagnostic test (Sagittal Index C7 + L3 and L3) values, and confidence interval are shown in Table 3. Again, it was not possible to predict trunk morphology due to facial sagittal plane morphology.

**Table 3:** Physical examination findings in facial sagittal morphology

Result	2 × 2 table		Sensitivity	Specificity	PPV	NPV	OR (95% CI)	+LR (95% CI)	−LR (95% CI)
Sagittal spinal posture	TP	FP							
	TN	FN							
Kyphosis line C7 + L3	69	87	44.2	60.7	16.7	85.9	1.23 (0.87–1.73)	1.13 (0.93–1.37)	0.92 (0.79–1.07)
	343	530							
L3	142	206	40.8	60.4	34.5	66.6	1.05 (0.86–1.37)	1.03 (0.88–1.20)	0.98 (0.88–1.09)
	270	411							

*T* true, *F* false, *P* positive, *N* negative, *PPV* positive predictive value, *NPV* negative predictive value

The association of facial sagittal morphology and sagittal spinal posture was estimated to have a point prevalence rate of 1.20 and 1.38% for C7 + L3 and L3, respectively. The possibility to predict a bad trunk sagittal posture due to the presence of an anomalous sagittal facial morphology had very low sensitivity, specificity, positive and negative predictive values, and likelihood ratios (Table 3).

## Discussion

The present study unrolled a large sample of 1029 healthy subjects evaluated through screening procedures to check the association between head and trunk asymmetries, suggestive of possible underlying bone deformities. For this reason, we chose significant cut-off to define scoliosis (7° ATR) and asymmetry (4° ATR). The prevalence of scoliosis and asymmetries in our sample, was small, any relationship between facial asymmetry and trunk asymmetry was found, and the same for sagittal facial morphology and sagittal spinal posture.

Kim et al. [14] found no apparent relation between the severity of scoliosis and facial form variations in idiopathic scoliosis patients. Various factors can determine the facial asymmetry, such as a distortion of the mandible, the maxilla, and other portions of the face can produce facial asymmetry and a distortion of the entire face.

The study did not focus on patients, but on general population. In fact, to eventually hypothesize a causal relationship, any possibly existing correlation should be found at the very early stage of diseases, rather than in already established deformities because the probability of secondary adaptations increases. Nevertheless, the study confirms the results of Kim in a totally different population.

Concerning the sagittal plane, many more relationships were found. The cervical spine is primarily responsible for the location of the head over the body as well as the level of horizontal gaze [32]. The natural curvature of the cervical spine maintains a lordotic shape because of the wedge-shaped cervical vertebrae and the need of compensation for the kyphotic curvature of the thoracic spine. Cervical lordosis may depend on the anatomy of the Cervical Thoracic Joint; the site at which lordosis of the cervical spine changes to kyphosis in the thoracic spine [33]. The sagittal alignment of the cranium and cervical spine may be influenced by the shape and orientation of the thoracic inlet to maintain a balanced, upright posture and horizontal gaze, similar to the relationship between the pelvic incidence and lumbar lordosis [34]. Each segment of the spine is related to the adjacent one and multiple significant correlations among them were found [35]. Moreover, the global sagittal alignment can be influenced by the sagittal shape of the cervical segment [36]. In fact subjects with a positive sagittal vertical axis (SVA) demonstrated an increase in cervical lordosis. This cervical adaptation to sagittal global alignment is a compensatory mechanism to maintain a horizontal gaze. Therefore, cervical lordosis can be considered, similar to thoracic kyphosis and lumbar lordosis, as an adaptive spinal segment to global alignment [33].

This could be linked to Dolphens et al. study [3], which demonstrated that whole-body sagittal alignment differs between healthy immature subjects with and without coronal plane TA, with significant associations between coronal plane TA and trunk lean, thoracic kyphosis and BMI. In particular, it is demonstrated that in healthy subjects, spinopelvic balance has a significant influence on cervical spine sagittal balance. These data support the hypothesis that a certain biomechanical loading of spinopelvic complex in the sagittal plane may predispose a child to develop a deformity in other planes [37].

According to the results, since trunk sagittal configuration does not correlate with facial sagittal morphology, the link found with cervical lordosis is not confirmed with the trunk.

Results do not deny any possible correlations between sagittal facial configuration and body posture for the cervical spine, even if we can conclude that such associations do not exist for the trunk and low back. In low degree asymmetries, postural compensatory mechanism may have minimized the effects of maxillofacial characteristics on the trunk, and viceversa [13].

The major limitations include the following: the screening procedures used that precluded a real study of the bones, even if they are well established in the literature for trunk asymmetries study; the cross-sectional design that suppose any causal relationship, even if this could be the first stage before going to a longitudinal approach; the low prevalence of scoliosis and asymmetries in our sample that led to study relatively few scoliosis subjects, but really representative of a general population.

## Conclusions

No correlation between TA and facial asymmetry was found as probably TA is correlated with spinopelvic balance and not with facial asymmetry. Strengths of this study include the wide sample representative of a normal adolescent population and the use of pre-definite significant cut-offs to define scoliosis and asymmetries [38, 39]. Considering the very well-known low prevalence of structural deformities, the need for a large sample is clear. In this view, the cross-sectional design is the most feasible way to assess a rare condition, as scoliosis can be considered in a population of healthy adolescent, even if it does not allow any cause–effect inferences. This is the first study of such a wide sample.

## Abbreviations

TA: trunk asymmetry
ATR: angle of trunk rotation
FBP: forward bending position
PPV: sensitivity, specificity, positive
NPV: negative predictive values
SVA: sagittal vertical axis
HRL: horizontal reference line
LL: lip line
STML: soft tissue midsagittal line
Pm: midpoint of pupil
